# From humanitarian aid to humanization: When outgroup, but not ingroup, helping increases humanization

**DOI:** 10.1371/journal.pone.0207343

**Published:** 2018-11-27

**Authors:** Thomas Davies, Kumar Yogeeswaran, Maykel Verkuyten, Steve Loughnan

**Affiliations:** 1 Department of Psychology, University of Canterbury, Christchurch, New Zealand; 2 Department of Psychology, University of Edinburgh, Edinburgh, Scotland; 3 Department of Interdisciplinary Social Science, Utrecht University, Utrecht, Netherlands; Middlesex University, UNITED KINGDOM

## Abstract

Dehumanization and infrahumanization involve decreasing the humanity attributed to others. Despite the existence of a large body of work on these topics, little is known about how to increase outgroup humanization. Across two experiments, we examined the effects of intergroup and intragroup helping on dehumanization and infrahumanization. In Study 1, we showed that news of an outgroup helping the ingroup after a natural disaster reduced infrahumanization, but not dehumanization. Reduced infrahumanization emerged regardless of the amount of aid given by the outgroup. By contrast, learning about ingroup helping the outgroup following a natural disaster did not decrease dehumanization or infrahumanization, regardless of amount of aid offered by the ingroup. Study 2 replicated and extended these findings by demonstrating that only intergroup helping by the outgroup to the ingroup reduced dehumanization. Intragroup helping, by either the ingroup or outgroup had no influence on outgroup perceptions. We report all measures, manipulations, and exclusions in each study. Implications of recent research on intergroup helping as a means to outgroup humanization are considered.

## Introduction

Each year, many nations worldwide give to victims of natural disasters within their own country or elsewhere in the world. Such helping happens in the form of governments and everyday citizens pooling funds together or offering human capital in wake of the disaster. How do news stories about such intergroup and intragroup helping influence the perceived humanization of the afflicted nation and the nation offering aid? The present research examines how both intergroup and intragroup helping following natural disasters influence humanization of ingroups and outgroups.

### (De-)humanization

Dehumanization is the process of denying humanity to others, a process which reduces moral concern, and can facilitate atrocious intergroup behaviour, including genocide in World War II when the Nazis compared Jews to vermin, or during the Rwandan genocide when Tutsis were compared to cockroaches [1]. Although the examples above represent the likening of others to animals (i.e., animalistic dehumanization), dehumanization can also manifest itself as mechanistic whereby others are seen as machine-like (i.e., mechanistic dehumanization) [2]. Various authors have highlighted that dehumanization not only occurs in these relatively blatant ways, but can also manifest in more subtle ways, such as infrahumanization—the denial of uniquely human characteristics or emotions to outgroups (for reviews, [2, 3]). Dehumanization has been shown towards women [4] the homeless [5], and ethnic others in a time of need [6, 7].

Despite an abundance of research on dehumanization, little is known about how to increase the humanity attributed to others. The limited research examining ways to increase humanization has provided participants with information about the target e.g., including a superordinate human identity [8]; the perceiver, e.g., manipulating feelings of power [9]; or the nature of intergroup relations. Most relevant to the present research is recent focus on the nature of intergroup relations as a means to promoting outgroup humanization. For example, in one study, imagined prosocial intergroup contact increased Italian children’s attribution of human emotions towards immigrants [10]. Children instructed to imagine interacting with an immigrant child and think about what nice things the participant could say showed increased humanization of the outgroup.

Other studies have directly manipulated intergroup helping to examine whether it changes the perceived humanity of the other. As dehumanization removes moral concern and justifies atrocious intergroup behavior, people can dehumanize others to justify the ingroup’s mistreatment of the outgroup ([1, 11, 12], or to justify failing to help the outgroup in times of need (e.g., following a natural disaster: [6, 7]). In relation to humanization, Saguy and colleagues theorized that, in order to justify the ingroup’s good deeds towards a dehumanized outgroup, people would humanize an outgroup if reminded of a time when the ingroup helped the outgroup [13]. In one study, Israeli participants read a short story about Israeli doctors (ingroup members) volunteering to help Palestinian children (outgroup members) in the Gaza strip, an area of contention between the two groups. Relative to controls, participants who read about the ingroup helping the outgroup showed an increase in humanization of the outgroup. A second study extended these findings by showing that news of a third party helping the outgroup did not increase the humanity attributed to the outgroup suggesting that ingroup helping in particular drives humanization of the other. Saguy and colleagues [13] argue in line with cognitive dissonance theory [14] that when the ingroup helps an outgroup, people may rationalize the ingroup’s act of prosocial behavior by increasing humanity attributed to that outgroup. However, when a third party helps the outgroup, people do not need to justify the third party’s behaviour, and do not increase the humanity attributed to the outgroup.

However, other work has demonstrated that knowledge of an outgroup helping a third party also reduces dehumanization of the outgroup that offered assistance [15]. Compared to a control condition, Spanish participants who read about Ethiopians prosocial behavior during a Somalian famine attributed more secondary emotions to Ethiopians. Delgado and colleagues [15] argue that outgroup helping may influence perceptions of outgroup humanity simply by priming prosociality.

### Current research

While previous research [13, 15] sheds some light on the influence of intergroup helping for outgroup humanization, it leaves several important questions unanswered. First, while Saguy and colleagues [13] examine intergroup helping in a conflict-ridden context, it is unclear if such findings would replicate in a non-contentious context, where the cause of the suffering is natural. Second, although Delgado and colleagues [15] suggest priming prosociality may be the mechanism behind why outgroup helping reduces dehumanization, it is not clear if that is truly the case. For example, would intragroup helping (e.g., outgroup helping its own people) elicit the same benefits of intergroup helping on humanization, or is helping others uniquely beneficial for outgroup humanization? Third, does the level of outgroup humanization depend on the amount of help offered to the victim? Previous research suggests that people experience greater attitude change as a function of the amount of effort expended [16]. In the context of the present research, this could mean that participants would attribute greater humanity toward the outgroup if the ingroup helps at a greater cost to itself, compared to when the help is minimal. By contrast, any amount of help from the outgroup to the ingroup may be seen as diagnostic of the helper’s underlying humanity, and therefore, might increase outgroup humanity regardless.

Across two studies, we examined these questions by testing the effects of intergroup and intragroup helping on both infrahumanization and dehumanization. In Study 1, we hypothesized that (1) news of an outgroup helping the ingroup would decrease both infrahumanization and dehumanization of an outgroup; (2) news of the ingroup helping the outgroup would also lead to less infrahumanization and dehumanization of the outgroup as a function of the amount of aid offered. In Study 2, we sought to replicate and extend the same expected findings by additionally testing (3) whether intragroup helping (i.e., knowledge of the ingroup helping itself, and the outgroup helping itself) would affect infrahumanization and dehumanization of the outgroup. We did not have directional predictions for our third hypothesis. It could be argued that both inter- *and* intra-group helping would promote greater outgroup humanization. Due to the organization and empathy required to instigate relief efforts, news of the outgroup helping itself after a natural disaster might lead observers to see the outgroup as more human compared to hearing of the natural disaster with no specific mention of help. Alternately, it is also possible that such an effect does not exist because people tend to expect that members of the same group normatively would help each other [17, 18]. Specifically, there are moral norms about being concerned about the welfare of one’s own group [19] and people apply the general rule “all individuals should help others of their own group” to both their own group and other groups [18]. Although refusal to help members of one’s own group invites disapproval, providing help is common and thus not very noteworthy [18]. This would mean that intragroup helping, or actions taken to help victims in one’s own country, may have no effect on outgroup humanity. In the present work, Americans represented the ingroup, while a pilot study helped determine an appropriate outgroup.

## Pilot study

### Method

The University of Canterbury Human ethics board approved this research. A total of 164 American adults (100 male; 64 female) were recruited from Crowdflower [20], an online platform very similar to Amazon’s Mechanical Turk. Sample size for the pilot (and the two subsequent studies), was determined a priori based on previous work in this area [6, 13] to yield at least 40 participants per cell. In the pilot study, however, each participant evaluated the humanness of six groups from a pool of Americans, Mexicans, Indonesians, Jordanians, Dijiboutians, Arabs, Thai, Pakistanis and Haitians using popular measures of animalistic and mechanistic dehumanization. To measure animalistic dehumanization we used a 4-item measure taken from Leidner, Castano, Zaiser, and Giner-Sorolla [21]; a sample question from the scale is “Pakistanis are typical of a backward culture". To measure mechanistic dehumanization we used a 4-item measure taken from Bastian and Haslam [22]; a sample question from the scale is “Pakistanis are mechanical and cold like they are robotic". Participants answered both measures using a scale anchored from 1 (*Strongly Disagree*) to 7 (*Strongly Agree*). For a full copy of the measures please see osf.io/ucfyv.

### Results

Of all the groups evaluated, Pakistan was chosen as the appropriate outgroup for several reasons. First, Pakistan was sufficiently dehumanized by Americans (see osf.io/ucfyv). Second, both America and Pakistan publically offered a similar amount of aid (relative to GDP) to the other following recent natural disasters. Third, Pakistan experienced a similarly impactful natural disaster to Hurricane Katrina with the 2010 flooding that caused a similar number of fatalities as Hurricane Katrina (approx. 2000).

## Study 1

We first examined how news of intergroup helping following the 2005 American disaster, Hurricane Katrina, or the 2010 Pakistan flooding, impacted Americans’ humanization of the outgroup (Pakistan). Additionally, we tested whether the amount of help offered following the natural disaster influenced the degree of humanization. Studies 1 and 2 contain a subsample of measures from a larger study that included individual differences variables not reported in the current work. For a full list of the measures see osf.io/ucfyv. There were no additional conditions to those reported in the present study.

## Method

The University of Canterbury Human ethics board approved this research.

### Participants

Three-hundred and eighteen participants completed the study. Fifty-five participants were removed from the study because they were either not US citizens, had missing data, or failed a basic manipulation check (i.e., ‘which nation did the disaster occur in?’), leaving a sample of 263 participants (*N* = 40–48 per cell) (147 female; *M*_age_ = 37 years, *SD* = 12.93). Those participants removed from analyses were evenly distributed across conditions. Participants were recruited from Crowdflower [20] and were paid US$2 for their time.

### Manipulations

Participants read one of six manipulations that were adapted from recent work examining dehumanization [13]. We used news stories that were based on actual events to manipulate the direction of help (America to Pakistan vs. Pakistan to America) and the amount of aid given (no aid vs. small amount of aid vs. large amount of aid).

#### Ingroup disaster

Half of the participants read a paragraph on Hurricane Katrina and its impact on Americans. In the control condition, the text ended after this paragraph. In the small and large aid conditions, participants read an additional paragraph about Pakistan donating either a small or a large amount of aid to the victims of Hurricane Katrina. Small and large aid conditions differed in terms of the number of doctors who volunteered (6 vs. 60), the amount of aid that was given by the country in US dollars (ostensibly 1% vs. 20% of the country’s humanitarian budget), and the amount of money donated by average citizens toward relief efforts ($50,000 vs. $500,000).

#### Outgroup disaster

The other half of the participants read about the 2010 Pakistan floods and its impact on Pakistanis. In the control condition, the text ended after this paragraph. In the small and large aid conditions, participants read an additional paragraph about America donating either a small or a large amount of aid to the victims of the Pakistan floods. We kept the amount of humanitarian aid and the total amount of money donated by average American citizens in the small and large aid conditions comparable to the ingroup disaster (US$500,000 vs. $25,000,000, ostensibly 1% vs. 20% of the country’s humanitarian budget).

### Measures

#### Infrahumanization

Participants were asked how strongly they believed Pakistanis would have felt both secondary and primary emotions following the disaster (ingroup or outgroup disaster). The emotions were seven negative secondary (α = .86; grief, sorrow, mourning, anguish, guilt, remorse and resentment; 1 = *Not at all*; 5 = *Extremely*), and seven negative primary emotions (α = .93; confusion, pain, distress, fear, panic, anger, and rage; 1 = *Not at all*; 5 = *Extremely*). These items were taken from previous work on infrahumanization and helping behaviour following a natural disaster [6].

#### Dehumanization

To assess participants’ dehumanization of Pakistanis, an 8-item measure (α = .86) was taken directly from work by Leidner and colleagues [21]. Participants were instructed to indicate how much they agreed with various statements regarding Pakistanis such as: “Some aspects of Pakistani life are typical of a backward culture". Responses were on a scale anchored from 1 (*Strongly Disagree*) to 7 (*Strongly Agree*), where higher numbers indicate greater outgroup dehumanization.

### Procedure

Participants first answered general demographic questions (including age, gender, nationality) before being randomly assigned to one of six conditions [2 (disaster: America or Pakistan) x 3 (aid: none, small, large)]. All participants then completed the dependent measures and the manipulation check before being debriefed and paid for their participation.

## Results

### Infrahumanization

See Table 1 for correlations between all variables in study 1. See Table 2 for means and standard deviations of all variables in study 1.

**Table 1:** Bivariate correlations between all variables in Study 1.

Measure	1.	2.
1. Secondary Emotions	-	
2. Primary Emotions	.869**	-
3. Dehumanization	.149*	.171*

*p < .05,

**p < .01

**Table 2:** Mean (SD) scores of infrahumanization and dehumanization in Study 1.

Disaster	Aid	Infrahumanization		Dehumanization
		Primary	Secondary	
Pakistan Disaster	Control	3.85 (1.21)	3.73 (0.91)	4.09 (1.16)
	Small Aid	4.05 (0.89)	3.61 (0.68)	3.86 (1.08)
	Large Aid	4.01 (.81)	3.79 (0.69)	3.91 (1.13)
American Disaster	Control	2.17 (1.05)	2.23 (0.93)	3.87 (0.89)
	Small Aid	2.04 (0.88)	2.70 (0.89)	3.61 (1.05)
	Large Aid	2.27 (0.98)	2.67 (0.76)	3.65 (1.02)

#### Ingroup disaster

Following Cuddy and colleagues [6], we analysed both secondary and primary emotions separately.

**Fig 1:**
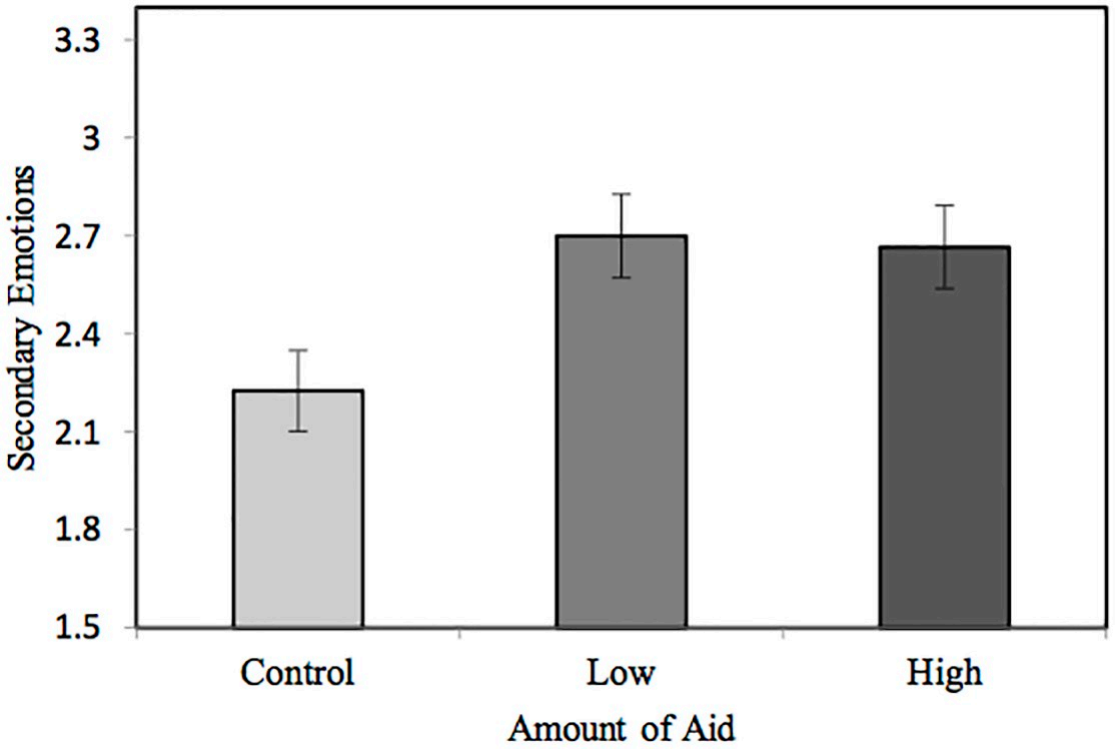
Mean (± 1 SE) level of secondary emotions attributed to Pakistanis by Americans after reading about Hurricane Katrina in Study 1.

In line with our hypotheses, a one-way ANOVA revealed a significant difference between conditions in attributions of outgroup *secondary emotions*, *F*(2,121) = 4.03, *p* = .020, $\eta_{p}^{2}=.062$. Post-hoc comparisons with Bonferroni adjustments revealed participants who read about Pakistan giving a small amount of aid attributed significantly more secondary emotions to Pakistanis than those participants who read about the disaster and no help, 95% CI [.02, .93]. In addition, participants who read about Pakistan giving a large amount of aid attributed marginally more secondary emotions to Pakistanis than no aid mentioned, 95% CI [-.01, .89]. However, there was no significant difference in secondary emotions between small and large aid conditions, 95% CI [-.43, .50]. These findings suggest that for small and large aid conditions compared to controls, the increase in secondary emotions attributed to the outgroup constitutes (marginally) reduced infrahumanization as shown in previous work [6] (see Fig 1).

A one-way ANOVA suggested there was no significant difference between conditions in attributions of outgroup *primary emotions*, *F*(2, 121) = 0.60, *p* = .552, $\eta_{p}^{2}=.010$.

#### Outgroup disaster

Contrary to our hypotheses, one-way ANOVA found no difference between conditions in attributions of outgroup *secondary emotions F*(2, 136) = 0.61, *p* = .547, $\eta_{p}^{2}=.009$ .

A similar one-way ANOVA suggested there was no difference between conditions in attributions of outgroup *primary emotions*, *F*(2, 136) = 0.57, *p* = .569, $\eta_{p}^{2}=.008$. Taken together, for those participants in the outgroup disaster conditions, we found no reduction in outgroup infrahumanization.

### Dehumanization

#### Ingroup disaster

In contrast to our hypotheses, a one-way ANOVA revealed that for participants who read about an ingroup disaster, there was no significant effect of aid on outgroup dehumanization, *F*(2, 121) = 1.90, *p* = .153, $\eta_{p}^{2}=.031$.

#### Outgroup disaster

A one-way ANOVA also showed that for those participants who read about an outgroup disaster, there was no significant effect of aid on outgroup dehumanization, *F*(2, 136) = 0.52, *p* = .595, $\eta_{p}^{2}=.008$.

## Discussion

When American participants read news of Pakistan helping America after Hurricane Katrina, they showed less infrahumanization compared to the control condition, but no differences in dehumanization of Pakistanis, when Pakistan offered small amounts of aid, and marginally when offering a large amount of aid. The difference in infrahumanization may be because participants saw the Pakistani response as an indication they experienced complex secondary emotions after the tragedy. However, news of America helping Pakistan following a disaster in Pakistan did not reduce outgroup infrahumanization, irrespective of the size of aid sent by the ingroup. This finding did not support our hypothesis, or that of Saguy and colleagues [13], and suggests that intergroup helping may not always impact on outgroup infrahumanization. It is interesting to note that there is a correlation between infrahumanization and dehumanization measures in this work. For example previous work has suggested that infrahumanization is so subtle that it can occur independently of negative evaluations of the outgroup [2]. In addition, Americans showed no change in their dehumanization of Pakistanis in any condition, a finding different from that of Saguy and colleagues [13] which specifically demonstrated an effect of intergroup helping on dehumanization. To be sure of this, we used a different dehumanization measure in Study 2, by opting to use the same measure used by Saguy and colleagues [13].

Despite evidence that outgroup helping after a natural disaster reduces infrahumanization, two important additional questions remain. First, is the effect of outgroup helping on humanization a result of realising that the outgroup is capable and willing to help, or is it specific to helping the *ingroup*? The former would reflect a change in group perception, the latter in intergroup relations. To test this question, Study 2 examined both intergroup helping (replicating Study 1) and intragroup helping (Americans helping themselves after a natural disaster in their own country, and Pakistani helping themselves after a natural disaster in their own country). Second, we wanted to examine if the findings of Study 1 would replicate in the context of a different ingroup disaster. Specifically, Hurricane Katrina is considered a controversial disaster by some Americans, as national relief efforts directed towards those most vulnerable (e.g., the poor, Black-Americans, and elderly) in the wake of Hurricane Katrina were deemed inadequate [6]. In order to examine the generalizability of the previous findings, we used Hurricane Sandy as the US disaster in Study 2.

## Study 2

The University of Canterbury Human ethics board approved this research.

## Method

### Participants

Four hundred and fifty-six participants completed the study on Crowdflower for US$2 [20]. In line with recommendations by Simmons, Nelson & Simonsohn [23] we ensured we had 50 participants per cell to get a more conservative estimate of our effect. Seventy-seven participants were removed from analyses because they were either not US citizens; there was missing data, or failed a basic manipulation check (‘which nation did the disaster occur in?’), leaving a sample of 379 American participants (201 female; *M*_age_ = 34years). Those participants removed from analyses were evenly distributed across conditions.

### Manipulations

Participants were randomly assigned to read one of six articles adapted from study 1.

#### Outgroup disaster

Half of the participants’ manipulation opened with a paragraph on the 2010 Pakistan floods and the impact it had on Pakistanis. In the control condition, the text ended after this paragraph, while in the US helping Pakistan condition, they read about aid offered by America similar to Study 1. In the intragroup helping condition, the prime was identical (to the above US helping Pakistan condition) except that participants read about average Pakistanis’ contributions to the relief efforts.

#### Ingroup disaster

The remaining participants read a paragraph about Hurricane Sandy (an ingroup disaster) and the impact it had on Americans (the ingroup). In the control condition, the text ended after this paragraph, while in the Pakistan helping US condition, they read about aid offered by Pakistan similar to Study 1. In the intragroup helping condition, the prime was identical (to the above Pakistan helping US condition) except that participants read about average Americans contributions to the relief efforts.

### Measures

#### Infrahumanization

The infrahumanization measures were identical to those used in study 1 (secondary emotions α = .90; primary emotions α = .93).

#### Dehumanization

In line with previous research [4, 13, 21] to assess dehumanization we got our measure from Saguy and colleauges [13]. We chose to use this measure instead of the one from Study 1 in order to ensure that the differences between the findings of Saguy and colleauges [13] and the present work are not a result of measurement differences. Saguy and colleauges [13] used mesaures aimed at their specific Israeli participants [24]; because our participants were American, we used dehumanization measures from the same study [24] which have been shown by American participants to best capture dehumanizaiton. Replicating Saguy and colleauges [13], participants were asked to imagine they meet someone from Pakistan, and to identify how much each of 3 positive uniquely human characteristics (calm, imaginative, and humble; α = .79) and 3 negative uniquely human characteristics (dominant, simple, and narrow-minded; α = .60) were typical of Pakistanis. Participants were also asked to identify how much each of 3 positive human nature characteristics (sympathetic, bold, and humorous; α = .66) and 3 negative human nature characteristics (defensive, stubborn, and aggresive; α = .78) were typical of Pakistanis. All responses ranged from 1 (*Not Typical At All*) to 6 (*Very Typical*), whereby lower scores equate to more dehumanization.

### Procedure

The procedure was identical to study 1. The study employed a 2 (country of disaster: Pakistan vs. USA) x 3 (aid: no aid vs. outgroup gives vs. ingroup gives) between-subjects design measuring infrahumanization and dehumanization.

## Results

### Infrahumanization

See Table 3 for means and standard deviations of infrahumanization in study 2; Table 4 for correlations between all variables in study 2; and Table 5 for means and standard deviations of dehumanization in study 2.

**Table 3:** Mean (SD) scores of infrahumanization in Study 2.

Disaster	Aid	Infrahumanization	
		Primary	Secondary
Pakistan Disaster	Control	3.53 (1.15)	3.41 (0.75)
	US Gives	3.84 (1.00)	3.66 (0.68)
	Pakistan Gives	3.67 (0.94)	3.67 (0.74)
American Disaster	Control	1.91 (1.03)	2.08 (0.92)
	US Gives	1.87 (0.98)	2.13 (0.88)
	Pakistan Gives	2.35 (0.93)	2.75 (0.78)

**Table 4:** Bivariate correlations between all variables in Study 2.

Measure	1.	2.	3.	4.	5.
1. Secondary Emotions	-				
2. Primary Emotions	.882**	-			
3. Mechanistic (Positive)	.198**	.133*	-		
4. Mechanistic (Negative)	.038	.077	.204**	-	
5. Animalistic (Positive)	.253**	.189**	.685**	.010	-
6. Animalistic (Negative)	.038	.070	.232*	.636**	.184**

*p < .05,

**p < .01

**Table 5:** Mean (SD) scores of dehumanization (mechanistic and animalistic) in Study 2.

Disaster	Aid	Mechanistic		Animalistic	
		Positive	Negative	Positive	Negative
Pakistan Floods	Control	3.36 (.74)	3.07 (0.93)	3.43 (0.82)	3.06 (0.89)
	US Gives	3.43 (.66)	3.31 (0.80)	3.40 (0.68)	3.13 (0.78)
	Pakistan Gives	3.48 (0.61)	3.12 (0.80)	3.51 (0.70)	3.07 (0.76)
Hurricane Sandy	Control	3.35 (0.70)	3.40 (0.72)	3.20 (0.86)	3.26 (0.67)
	US Gives	3.49 (0.70)	3.38 (0.88)	3.50 (0.74)	3.36 (0.73)
	Pakistan Gives	3.48 (0.69)	3.15 (0.68)	3.66 (0.73)	3.12 (0.71)

#### Ingroup disaster

**Fig 2:**
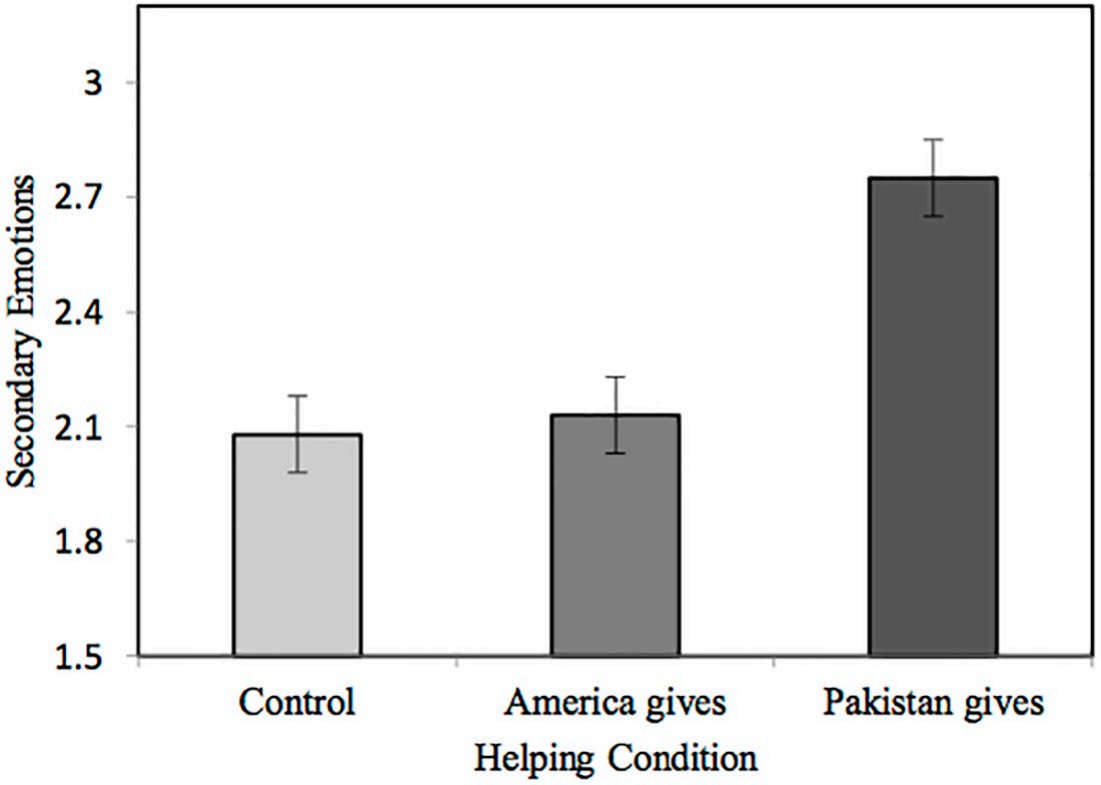
Mean (± 1 SE) level of secondary emotions attributed to Pakistanis by Americans after reading about Hurricane Sandy in Study 2.

In line with our hypotheses, a one-way ANOVA revealed a significant difference between conditions in attributions of outgroup *secondary emotions*, *F*(2, 180) = 11.65, *p* < .001, $\eta_{p}^{2}=.115$. Post-hoc comparisons with Bonferoni adjustments revealed participants who read about Pakistan helping America attributed significantly more secondary emotions to Pakistanis than participants who read about Americans helping themselves, 95% CI [.25, 1.00], or those who read about the ingroup disaster with no mention of aid to America, 95% CI [.30, 1.05]. In addition, there was no difference in attributions of outgroup secondary emotions between participants who read about Americans helping themselves and those who read about no aid being given to America, 95% CI [-.33, .43]. Taken together, for participants who read about the outgroup helping the ingroup, the larger attribution of secondary emotions constitutes reduced outgroup infrahumanization (see Fig 2).

A one-way ANOVA suggested there was a difference in attributions of outgroup *primary emotions F*(2, 180) = 4.53, *p* = .012, $\eta_{p}^{2}=.048$. Post hoc comparisons with Bonferoni adjustments showed participants who read about the outgroup helping the ingroup also attributed more primary emotions to the outgroup compared to those who read about the ingroup helping the ingroup, 95% CI [.05, .91], or no help mentioned, 95% CI [.02, .87].

#### Outgroup disaster

In contrast to our hypothesis, a one-way ANOVA showed no significant difference between conditions in attributions of outgroup *secondary emotions F*(2, 192) = 2.79, *p* = .064, $\eta_{p}^{2}=.028$.

A one-way ANOVA showed no significant difference between conditions in attributions of outgroup *primary emotions F*(2, 192) = 1.48, *p* = .231, $\eta_{p}^{2}=.015$.

### Dehumanization

#### Ingroup disaster

Following the analysis strategy of Saguy and colleauges [13] we ran a mixed model ANOVA for participants who read about an ingroup disaster, with help condition as a between-subjects factor and characteristic type (mechanistic vs. animalistic) and valence (positive vs. negative) as within-subjects factors. The three-way interaction between aid condition, characteristic type, and valence was not significant, *F*(2, 174) = 1.23, *p* = .288, $\eta_{p}^{2}=.014$. There was a main effect of valence, such that participants attributed more positive traits to the outgroup (*M* = 3.44, *SE* = 0.05) than negative traits (*M* = 3.28, *SE* = 0.05), *F*(1, 174) = 6.39, *p* = .012, $\eta_{p}^{2}=.035$. The analysis revealed no difference in characteristics, such that overall, participants attributed the same level of human nature (*M* = 3.38, *SE* = 0.04) and human uniqueness characteristics (*M* = 3.35, *SE* = 0.04), *F*(1, 174) = 0.57, *p* = .451, $\eta_{p}^{2}<.003$. There was a significant interaction between aid condition and type of humanization, *F*(2, 174) = 3.65, *p* = .028, $\eta_{p}^{2}=.040$. To break down the interaction we computed a multivariate ANOVA with both types of humanization (mechanistic vs animalistic) as outcome variables. The results revealed no significant difference between aid conditions for either mechanistic *F*(2, 174) = 0.67, *p* = .521, $\eta_{p}^{2}=.007$, or animalistic dehumanization *F*(2, 174) = 2.03, *p* = .135, $\eta_{p}^{2}=.023$.

#### Outgroup disaster

We ran an identical mixed model ANOVA for participants who read about the outgroup disaster. Similarly, the three-way interaction was not significant, *F*(2, 186) = 0.13, *p* = .879, $\eta_{p}^{2}=.001$. There was a significant main effect of valence such that participants attributed more positive traits to the outgroup (M = 3.44, SE = 0.05) than negative traits (*M* = 3.13, *SE* = 0.06), *F*(1, 186) = 23.59, *p* < .001, $\eta_{p}^{2}=.113$. The analysis revealed no difference in traits, such that overall, participants attributed the same level of human nature (*M* = 3.30, *SE* = 0.04) and human uniqueness characteristics (*M* = 3.27, *SE* = 0.04), *F*(1, 186) = 0.49, *p* = .486, $\eta_{p}^{2}<.003$. There was also no main effect of condition, *F*(2, 186) = 0.44, *p* = .643, $\eta_{p}^{2}=.005$. Additionally, there was no interaction between aid condition and type of humanization, *F*(2, 186) = 1.07, *p* = .347, $\eta_{p}^{2}=.011$.

## Discussion

Study 2 examined the impact of both intergroup and intragroup helping after an ingroup and outgroup natural disaster on the perceived humanization of the outgroup. The results once again provided evidence that participants who read about an outgroup helping the ingroup following a natural disaster showed less infrahumanization of the outgroup. In line with Study 1, the effect of an outgroup helping the ingroup only had an impact on infrahumanization but not dehumanization of Pakistanis. Knowledge of fellow Americans helping after the American natural disaster had no effect on humanization of Pakistanis, suggesting again, that helping after a natural disaster does not uniformly prime humanization of others, addressing previous speculation in the literature [15]. However, when participants read about the Pakistani floods, regardless of whether relief efforts were led by America (similar to Study 1) or Pakistan itself (intragroup helping), there was no change in the extent to which Pakistanis were infrahumanizned or dehumanized (using the same measure as Saguy and colleagues [13]). This is in contrast with Saguy and colleagues’ [13] work showing that news of the ingroup helping an outgroup can improve humanization. It is interesting to note that the correlation between secondary emotions and dehumanization was far stronger for positive traits than for negative ones. In addition, we found a valence effect for our dehumanization measure whereby participants attributed overall more positive than negative traits to the outgroup.

## General discussion

The present research examined how news stories about intergroup and intragroup helping following natural disasters influence infrahumanization and dehumanization of the outgroup. In Study 1, American participants who read a news story about Hurricane Katrina, and were told that Pakistan had offered aid after the disaster showed a decrease in infrahumanization of Pakistanis (relative to a no-information control) when Pakistan offered a small amount of aid, and marginally when offering a large amount of aid. However, these effects did not carry over to outgroup dehumanization, a more blatant measure. In contrast to previous work [13], when American participants read a news story about the 2010 Pakistan floods, they showed no change in their humanization of Pakistanis regardless of whether the US had offered large or small amounts of aid in response to the disaster. In Study 2, we examined the effects of both intergroup and intragroup helping on outgroup humanization using a different ingroup disaster (Hurricane Sandy) and a different dehumanization measure. Data revealed that news of Pakistan helping the US after Hurricane Sandy, replicating Study 1, decreased the infrahumanization, but not dehumanization of Pakistanis, relative to when no information on helping was made salient or when intragroup helping was made salient. However, news of the 2010 Pakistan floods and relief given by Americans did not influence outgroup infrahumanization or dehumanization and neither did reading about intragroup helping in response to their own natural disaster. Taken together, across two studies we found no evidence that ingroup helping improves outgroup humanization, and consistent evidence that an outgroup helping the ingroup can be a positive humanization strategy, at least in the context of natural disasters.

### Theoretical contribution

These findings are inconsistent with Saguy and colleagues [13] who found that reading about the ingroup helping the outgroup lead to more humanizing perceptions of the outgroup. However, it is noteworthy that Saguy and colleauges [13] only reduced mechanistic dehumanziation in one of their two studies; it could be argued that while secondary emotions are considered uniquely human by some psychologists, infrahumanization might also reflect the mechanistic side of dehumanization whereby those who are not attributed secondary emotions are seen as more cold and robotic than others, much like those who are mechanistically dehumanized. Nevertheless, there are at least two notable differences between the present work and that of Saguy and colleagues [13]. First, Saguy and colleagues [13] examined humanization of outgroups in a human-caused disaster, whereas the current work involves helping in response to a natural disaster. This may be important as a natural disaster may activate social categorization at the level of shared humanity, rather than national or religious groups. Secondly, the intergroup context of the two studies may be another important factor underlying the differences in the findings. Saguy and colleagues [13] conducted their work in the context of the Gaza strip, a conflict-ridden region, whereas the present research involved two nations without a direct history of conflict. As Saguy and colleagues [13] speculate, the benefits of intergroup help may be less pronounced in a non-violent context, and it may be that our findings reiterate that speculation. In particular, for the current study, it may be that infrahumanization was reduced and dehumanization was not because infrahumanization is a more subtle measure and is able to capture more subtle effects.

The current work advances a limited body of experimental work which outlines the parameters for reducing outgroup dehumanization. While previous work has demonstrated that outgroup humanization can be promoted via different means (e.g., imagining positive outgroup interactions [10]; having complex descriptions of the outgroup’s identity [8, 25]) the present work adds to the literature by showing that simply priming prosocial behaviour alone does not increase humanization. Intragroup helping is expected of social groups as they are perceived as fundamental to social living [22], therefore, providing ingroup help is common and does not promote changes to outgroup humanization [18]. Instead, an outgroup helping the ingroup (as observed here) or a third party [15] is more noteworthy and therefore increases the perceived humanity attributed to the other. Delgado and colleagues [15] work suggests that the mechanism between outgroup helping and humanization may be one of prosocial priming. However, we find that priming participants with intragroup helping does not improve outgroup humanization. The current work suggests that in this non-violent context [13] it is outgroup helping of an intergroup nature that yields positive humanization strategies, not intra-outgroup helping nor ingroup helping the outgroup. This is a positive finding, in that this is one of only a few studies showing an increase in outgroup humanization. However, a possible mechanism for understanding why ingroup to outgroup helping did not decrease dehumanization is because of group differences in power. Recent work by Nadler [26] argues that intergroup helping can backfire when there is a power difference between the two groups (such as there is between the US and Pakistan), as the powerful group helping the less powerful group can underline the low status group’s inferiority, and potentially their humanness. Related to the current work, upon finding out that one’s group has been helped by a lower status group may involve conceding ground to that outgroup and seeing them as more equal, perhaps more powerful, and consequently more human. Future research on intergroup helping may therefore benefit from exploring the mediating role of feelings of power, and the moderating individual differences that may accompany such a mechanism. Regarding intragroup helping not yielding an increase in secondary emotions, it is likely that such helping is normative and expected, and thus not worthy of improved preceptions of humanness. An additional mechanism is that intragroup helping might not necessarily lead to an increase in the attribution of moral value to the outgroup [27, 28]. Considering these limitations, future research on intragroup helping would benefit from exploring normative expectations, and changes in the moral value attributed to others as a possible mediator of outgroup humanness.

The current findings reveal that the outgroup-to-ingroup helping alleviated only infrahumanization, and did not reduce dehumanization. It may be that our dehumanization measures allowed participants to maintain the perspective that the outgroup is backwards in some ways (e.g. patriarchal gender relations), but attribute them increased mental capacity in other areas (e.g. complex human emotions). This is quite likely because of the difference in blatancy between infrahumanization and dehumanization. Haslam and Loughnan [2] argued that infrahumanization is so subtle that it can occur independently of other forms of dehumanization. With respect to the current findings, it may be that our manipulation was able to move infrahumanization, but not dehumanization of the outgroup.

In addition, we also found a valence effect for the dehumanization measure used in Study 2, whereby participants attributed more positive than negative human traits to the outgroup. What’s more, secondary emotions were positively associated with positive (mechanistic and animalistic) traits, and not correlated with negative (mechanistic and animalistic) traits. While speculative, it may be that for participants it is more forgivable to deny positive traits than to attribute negative ones, and also easier to increase positive traits to the outgroup than to reduce negative attributes of that outgroup. Therefore, it is important for future research to carefully choose the manipulations and measures used for humanization interventions, especially in a non-violent intergroup context.

Finally, while the current research adds important contributions to the literature on rehumanization strategies, we did not measure any eventual implications of respondents evaluation behavior. That is, it is unknown whether the attribution of greater secondary emotions to the outgroup would translate to real-world behavioral changes. Future work would greatly benefit from examining the behavioral consequences of such intergroup helping.

### Conclusion

The current work adds to the small field of humanization by identifying a novel scenario where attributions of outgroup humanity are increased. We suggest our findings complement and extend existing work on intergroup relations and humanization strategies [13, 15]. Across two studies, we found no evidence that news of an outgroup’s plight following a natural disaster coupled with help from the ingroup impacts the humanity attributed to that outgroup. However, across both studies, we found novel evidence that news of an outgroup helping the ingroup following a natural disaster can improve perceptions of outgroup humanity, independently of the amount of help offered.
